# Supplementary material for: From Burst to Sustained Release: The Effect of Antibiotic Structure Incorporated into Chitosan-Based Films
Source: Antibiotics (Basel). 2024 Nov 6;13(11):1055. doi: 10.3390/antibiotics13111055 (PMC11591004; doi:10.3390/antibiotics13111055)
Supplement: Supplementary file 1 [file antibiotics-13-01055-s001.zip › antibiotics-3273231-supplementary.pdf]

Article

From Burst to Sustained Release: The Effect of Antibiotic Structure Incorporated into Chitosan-Based Films

Nathália F. Sczesny [1,†], Helton J. Wiggers [1,†], Cecilia Z. Bueno [1], Pascale Chevallier [2,*], Francesco Copes [2] and Diego Mantovani [1,2,*]

[1] Laboratory for Biomaterials and Bioengineering (LBB-BPK), Associação de Ensino, Pesquisa e Extensão BIOPARK, Max Planck Avenue, 3797, Building Charles Darwin, Toledo 85919-899, PR, Brazil; nsczesny@outlook.com (N.F.S.); helton.wiggers@bpkedu.com.br (H.J.W.); cecilia.bueno@bpkedu.com.br (C.Z.B.)

[2] Laboratory for Biomaterials and Bioengineering (LBB-UL), Canada Research Chair Tier I, Department of Min-Met-Materials Engineering & Division Regenerative Medicine of CHU de Quebec Research Center, Laval University, Quebec City, QC G1V 0A6, Canada; francesco.copes.1@ulaval.ca

* Correspondence: pascale.chevallier@crchudequebec.ulaval.ca (P.C.); diego.mantovani@gmn.ulaval.ca (D.M.)

† These authors contributed equally to this work.

Abstract: Background/Objectives: Medical devices are susceptible to bacterial colonization and biofilm formation, which can result in severe infections, leading to prolonged hospital stays and increased burden on society. Antibacterial films have the potential to assist in preventing biofilm formation, thereby reducing administration of antibiotics and the emergence of antibiotic-resistant strains. In a previous study, a chitosan-based matrix crosslinked with tannic acid and loaded with gentamicin was reported. In this study, five different antibiotics (moxifloxacin, ciprofloxacin, trimethoprim, sulfamethoxazole or linezolid) were loaded into these chitosan-based films, and their impact on the release behavior carefully assessed. **Methods:** The samples were characterized according to their thickness, swelling, and mass loss in phosphate-buffered saline (PBS), as well as by morphology using scanning electron microscopy (SEM) and optical phase contrast microscopy. Antibiotic release over time was quantified in PBS by high-performance liquid chromatography (HPLC). Antibacterial activity was investigated by disk diffusion test and antibiotic release over time. Finally, the cytotoxicity of the samples was assessed with human dermal fibroblasts. **Results:** The obtained results differed significantly, especially regarding the antibiotic release time and antibacterial activity, which varied from one day to six months, enabling classification of the films from burst/transient to prolonged release. The films also showed antibacterial features against bacteria mostly present in medical devices and displayed to be non-cytotoxic. **Conclusion:** In conclusion, it was demonstrated that the antibiotics structure significantly alters the release kinetics, and that by carefully selecting the antibiotic, the consequent release can be tuned. This approach yielded films that could be used for potentially-scalable release in antimicrobial coatings specific to medical devices, aiming to reduce biomaterial associated infections (BAIs).

Keywords: Antibacterial film; Sustained drug delivery; Medical devices; Structure-property relationships.

Additional information to *2.1. Antibiotic release over time*

Table S1. Physicochemical propertied of the antibiotics structures

Antibiotics	Num. heavy atoms	Fraction Csp³	Nº rotatable bonds	Nº H-bond acceptors	Nº H-bond donors	TPSA[1] Å²	Log $P_{o/w}$ (iLOGP)[2]
Moxifloxacin	29	0.52	4	5	1	91.21	2.86
Ciprofloxacin	24	0.41	3	4	1	81.98	1.99
Trimethoprim	21	0.29	5	4	3	106.76	2.21
Sulfamethoxazole	17	0.10	3	5	1	94.57	1.62
Llinezolid	24	0.50	5	5	1	71.11	2.44

1 TPSA – Topological Surface Polar Area; 2 Log $P_{0/w}$ partition coefficient octanol/water calculated by iLOGP – Data obtained from SwissADME [1].

R^2, MSC and AIC are the main parameters to identify dissolution models. R^2 determines how well an independent variable predicts the outcome of a dependent variable; the closer to one, the better, shown in the main text Table 3. AIC (Akaike Information Criterion) depends on the magnitude of the data and the number of data points, the lower the AIC, the better. MSC depends on the observed and the predicted data, and the largest the MSC, the better, shown in Tabel S2. [2]

Table S2: Main parameters that determine a good fit of release on the mathematical models.

	Ciprofloxacin			Moxifloxacin			Trimethoprim			Linezolid			Sulfamethoxazole		
**Model**	**R^2**	**MSC**	**AIC**	**R^2**	**MSC**	**AIC**	**R^2**	**MSC**	**AIC**	**R^2**	**MSC**	**AIC**	**R^2**	**MSC**	**AIC**
Korsmeyer-Peppas	0.96	2.88	11.86	0.99	5.86	-20.24	0.96	3	58.24	NA	NA	NA	0.77	1.17	87.04
Weibull	0.97	3.14	8.42	0.99	6.07	-23	0.99	4.44	39.57	NA	NA	NA	0.99	4.72	40.92
Zero order	0.85	1.95	24	0.94	2.74	20.4	-0.3	-0.16	99.45	-2.35	-1.21	134.61	-4.26	-1.8	125.8
First order	0.86	2.03	22.96	0.95	2.93	17.93	0.35	0.33	92.89	-0.05	-0.2	120.57	-1.08	-0.88	113.83
Hopfenberg	0.86	1.87	24.97	0.94	2.77	19.95	0.35	0.18	94.89	NA	NA	NA	-1.09	-1.04	115.83
Higuchi	0.92	2.37	18.47	0.95	2.89	18.36	0.83	1.69	75.32	-0.09	0.06	117.94	-0.97	-0.83	113.05

Additional information to *2.3 Antibacterial activity*

The disk diffusion, also known as Kirby-Bauer test [3] as performed to check the antimicrobial activity of the samples. Six bacteria were used - *Escherichia coli, Enterococcus faecalis, Staphylococcus aureus, Staphylococcus epidermidis, Pseudomonas aeruginosa* and *Klebsiella pneumoniae*. Firstly, the bacteria were taken from frozen stocks and thawed to room temperature. Approximately 1×10^8 CFU/ml were spread with a Drigalski spatula onto 15 cm Petri dishes coated with fresh sterile Mueller–Hinton agar. Film samples (cut into a 6 mm-diameter disk) (standard NCCLS) were sterilized with UV irradiation at 254 nm for 15 min on each side, and then placed on Petri dishes containing the bacteria.

Paper disks impregnated with antibiotic were used as positive control and paper discs without antibiotic were used as negative control. The dishes were incubated at 37°C for 24h in an inverted position. Afterwards, the inhibition zones were measured using a digital pachymeter in three different positions [4,5]. Experiments were carried out in at least duplicates for each bacterium. The results for the antibiotic-loaded films are summarized in Table S3 and the results for the antibiotic positive controls in Table S4.

Table S3: Disk diffusion halos diameter of different antibiotics positive controls against bacteria frequently found in BAI.

	E. coli (mm)	***K. pneumoniae*** (mm)	***P. aeruginosa*** (mm)	***S. epidermidis*** (mm)	***S. aureus*** (mm)	***E. faecalis*** (mm)
Positive controls						
MOX	27.1 ± 1.53	30.4 ± 2.69	25.7 ± 1.87	23.7 ± 1.56	31.3 ± 0.53	26.0 ± 1.48
CIP	25.6 ± 3.16	33.7 ± 0.14	37.5 ± 0.74	30.5 ± 0.35	25.9 ± 0.86	23.6 ± 16.90
TMP	25.6 ± 0.79	30.4 ± 0.42	0	27.9 ± 0.14	22.8 ± 0.06	27.6 ± 0.67
SMX	22.6 ± 0.45	22.2 ± 2.05	0	0	10.4 ± 2.65	11.9 ± 16.9
LNZ	19.9 ± 1.37	18.9 ± 0.57	0	27.3 ± 0.64	27.1 ± 1.03	26.2 ± 0.45

The MIC test was carried out with the five antibiotics (trimethoprim, moxifloxacin, ciprofloxacin, linezolid and sulfamethoxazole) against six bacteria (*S. aureus, S. epidermidis, E. coli, P. aeruginosa, K. pneumoniae* e *E. faecalis*), in triplicates. The antibiotics concentrations were selected according to each bacterium susceptibility, and 100 μL of each solution were placed in 96-well culture plates and mixed with 100 μL of bacteria stock solution at 1×10^6 CFU/mL. Plates were incubated at 35°C and 200 rpm for 8 hours, except *P. aeruginosa*, which was incubated for 24 hours. Afterwards, the plates were analyzed in a microplate reader at 600 nm.

Table S4: Minimum Inhibitory Concentration (MIC) for the antibiotics against bacteria frequently found in BAI.

	Bacteria	CIP	MOX	LNZ	SMX	TMP
Gram +	*S. aureus* ATCC 6538	0.25	0.5	1	4	0.5
	S. epidermidis ATCC 12228	0.25	0.06	0.125	4	0.03
	E. faecalis ATCC 29212	0.25	0.125	1	8	0.06
Gram −	*K. pneumoniae* ATCC 10031	1	0.015	16	1	0.25
	E. coli ATCC 8739	0.015	0.03	64	1	0.125
	P. aeruginosa ATCC 9027	0.06	0.5	64	8	16

References

1. Daina, A.; Michielin, O.; Zoete, V. SwissADME: A Free Web Tool to Evaluate Pharmacokinetics, Drug-Likeness and Medicinal Chemistry Friendliness of Small Molecules. *Sci. Rep.* **2017**, *7*, 42717, doi:10.1038/srep42717.

2. Zhang, Y.; Huo, M.; Zhou, J.; Zou, A.; Li, W.; Yao, C.; Xie, S. DDSolver: An Add-In Program for Modeling and Comparison of Drug Dissolution Profiles. *AAPS J.* **2010**, *12*, 263–271, doi:10.1208/s12248-010-9185-1.

3. Hudzicki, J. Kirby-Bauer Disk Diffusion Susceptibility Test Protocol Available online: https://asm.org/protocols/kirby-bauer-disk-diffusion-susceptibility-test-pro (accessed on 3 September 2024).

4. NCCLS *Methods for Dilution Antimicrobial Susceptibility Tests for Bacteria That Grow Aerobically-Sixth Edition. Document M7-A6. Wayne, Pa*; 2003; ISBN 1562384864.

5. Matuschek, E.; Brown, D.F.J.; Kahlmeter, G. Development of the EUCAST Disk Diffusion Antimicrobial Susceptibility Testing Method and Its Implementation in Routine Microbiology Laboratories. *Clin. Microbiol. Infect.* **2014**, doi:10.1111/1469-0691.12373.

Disclaimer/Publisher's Note: The statements, opinions and data contained in all publications are solely those of the individual author(s) and contributor(s) and not of MDPI and/or the editor(s). MDPI and/or the editor(s) disclaim responsibility for any injury to people or property resulting from any ideas, methods, instructions or products referred to in the content.
